# Purely ultrasonic enzyme extraction from activated sludge in an ultrasonic cleaning bath

**DOI:** 10.1016/j.mex.2017.07.003

**Published:** 2017-07-18

**Authors:** Mario Plattes, Christian Koehler, Tom Gallé

**Affiliations:** Luxembourg Institute of Science and Technology (LIST), Environmental Research and Innovation (ERIN), Site de Belvaux, 41, rue Brill, L-4422 Belvaux, Luxembourg

**Keywords:** Enzymes, Extraction, Ultrasound

## Abstract

Enzymes are important in biological wastewater treatment systems, since they are responsible for breakdown of macro- and micropollutants, thereby making the pollutants available for metabolism. Enzyme activity has been investigated in particular in activated sludge processes, since the activated sludge technology is the most important and widely spread wastewater treatment technology used today. Various methods have been used to extract enzymes from activated sludge in order to measure their activity, these include stirring with additives like detergents and cation exchange resins, ultrasonication (with probes) and combinations of the three [1], [2], [3]. In this article we describe a method for purely ultrasonic enzyme extraction from activated sludge using low power ultrasound generated by an ultrasonic bath and no additives. The method essentially consists of:

• Sonication of the sludge sample using a glass beaker and an ultrasonic bath.
• Filtration of the sample in order to obtain the enzyme extract.
• Measurement of enzyme activity by fluorescence spectrometry using a substrate that yields a fluorescent product.

## Graphical abstract

**Figure:**
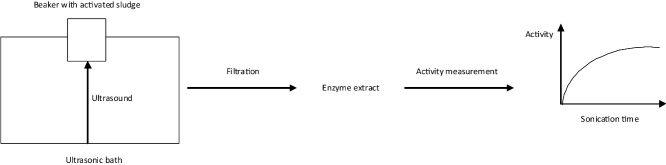

## Methods detail

### Activated sludge sampling and TSS/VSS measurement

An activated sludge sample (1 l) was taken from the aeration tank of the municipal wastewater treatment plant Petange (50 000 PE) in Luxemburg and transported to the laboratory within 30 min. Half of the sample was directly subjected to enzyme extraction. The other half was aerated over night using an aeration pump (SCHEGO M2K3) and subsequently subjected to enzyme extraction. Total suspended solids (TSS) and volatile suspended solids (VSS) were determined. In order to do so the sludge sample was filtered using glass fiber filters (1 μm pore size, PALL). The residue was dried over night at 105 °C in a drying oven (Memmert UN 110) and subsequently incinerated at 550 °C for 1.5 hours in a muffle furnace (Nabertherm). The weights were recorded.

### Ultrasonic enzyme extraction

An activated sludge sample (50 ml) was centrifuged for 5 min at 3000 rpm (Thermo Scientific SL 16 Centrifuge). The sediment was resuspended to original volume (50 ml) with sodium chloride solution (0.14 mol/l, Merck). Part of the sample (40 ml) was than sonicated in an ultrasonic bath (VWR Ultrasonic Cleaner USC-TH, 45 kHz, 180 W) using a glass beaker (100 ml) and various sonication times (0, 2.5, 5, 10, 15, and 20 min). The ultrasonic power in the beaker was determined by disequilibrium calorimetry described elsewhere (submitted). The ultrasonic power in the beaker was found to be 5.71 W. After sonication, the sludge was filtered using syringes (TERUMO) and membrane filters (0.45 μm, VWR). This gave the enzyme extracts.

### Enzyme assay

Esterase activity was measured by fuorescence spectroscopy using fluoresceine diacetate (Sigma-Aldrich) as substrate according to the method described by Obst [4]: The substrate solution was prepared by dissolving 10 mg fluoresceine diacetate in 5 ml ethylenglycol-monomethylether (SERVA). This solution was then diluted (1:2) with deionized water, which gave the final substrate solution. For the essay 200 μl of enzyme extract were pipetted into 3 cavities of a microtiter plate (Perkin Elmer) and 10 μl of substrate solution were added. For the blank 10 μl of substrate solution were added to 200 μl sodium chloride solution (0.14 mol/l, Merck). For the photometric blank 10 μl of substrate solution were added to 200 μl sodium chloride solution. A standard solution was prepared by dissolving 10 mg fluorescein sodium (Sigma-Aldrich) in 100 ml deionized water. An aliquot of 0.1 ml was diluted to 100 ml with deionized water (final concentration 0.1 mg/l). For the standards 10, 40, 70, 100, 130, 160, and 200 μl of standard solution were filled up to 200 μl with deionized water and 10 μl of substrate solution were added. The pH of all cavities was adjusted to 7.5 by addition of 30 μl HEPES-buffer (CALBIOCHEM). An overview of the microtiter plates preparation is given in Table 1. The excitation wavelength of the fluorescence spectrometer (Perkin Elmer LS55) was set to 485 nm and the emission wavelength was set 538 nm respectively. The resulting progress curves and enzyme activities were evaluated in Excel.

**Table 1:** Preparation of microtiter plates.

Cavity name	Content
Sample	200 μl enzyme extract
	10 μl substrate
	30 μl HEPES
Blank	200 μl enzyme extract
	10 μl sodium chloride
	30 μl HEPES
Photometric blank	200 μl sodium chloride
	10 μl substrate
	30 μl HEPES
Standard	10, 40, 70, 100, 130, 160, 200 μl standard
	190, 160, 130, 100, 70, 40, 0 μl water
	10 μl substrate
	30 μl HEPES

### Evaluation

**Fig. 1:**
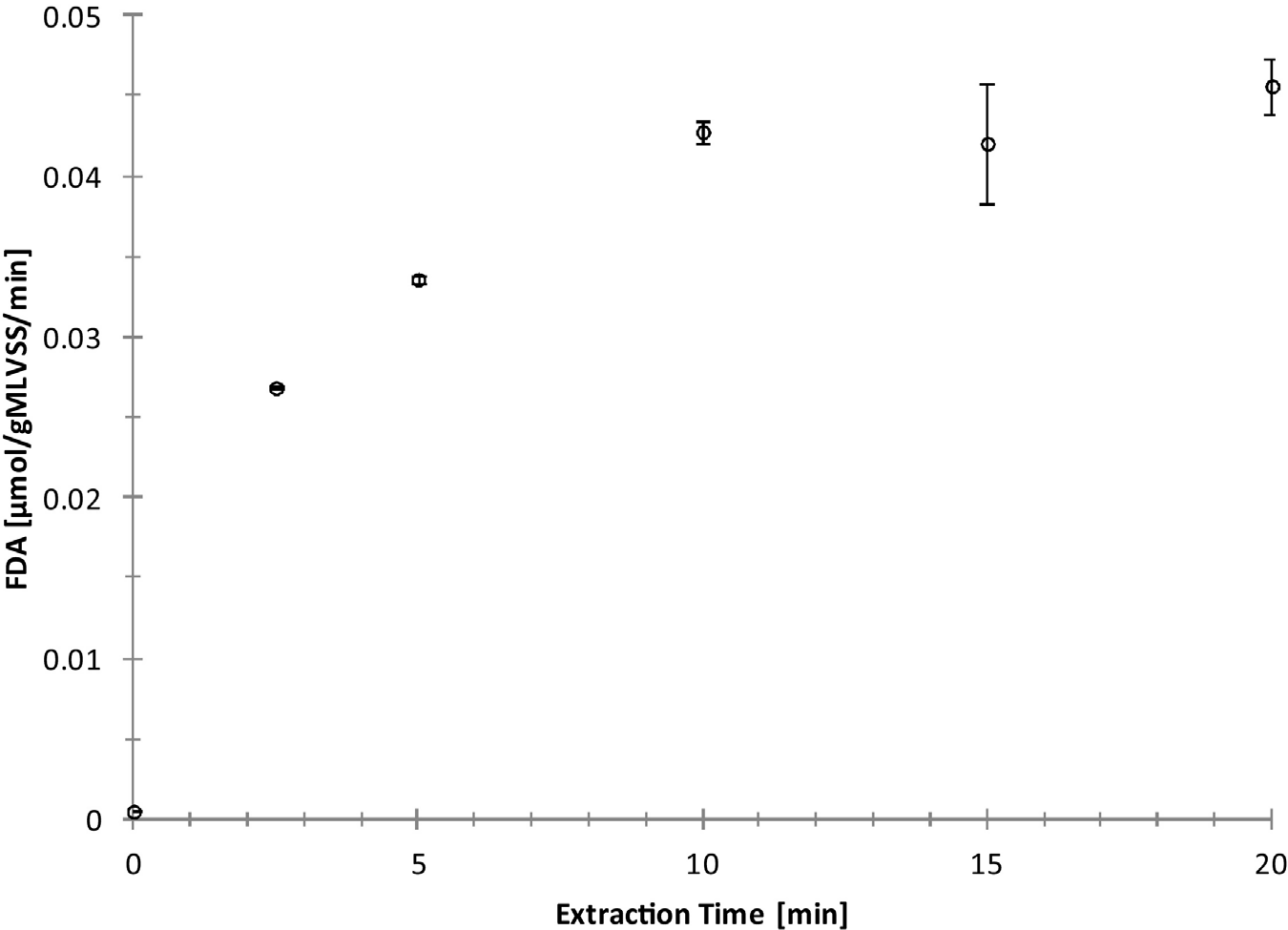
The effect of sonication time on FDA activity in the enzyme extracts using fresh activated sludge. The error bars show standard deviation resulting from triplicate activity measurement of the enzyme extract and can hardly been seen for the first three points.

Esterase activity in the enzyme extracts increased with increasing sonication time up to a sonication time of 10 min (Fig. 1). Longer sonication up to 20 min did not significantly increase the activity of the enzyme in the extract. The enzyme activity in the extract is thus a function of the ultrasonic power transferred over a certain time period to the activated sludge. The maximum activity was reached after 10 min sonication time. We suspect that the sonication time required to reach maximum enzyme activity in the extract is a function of the ultrasonic power of the system, i.e. it will be less when the ultrasonic power is bigger, e. g. when using a more powerfull ultrasonic probe. Further it was found that storage of the enzyme extracts in the fridge for 1 and 2 days significantly reduced enzyme activity by 30 and 87% respectively. Aeration of activated sludge to endogenous state prior to enzyme extraction reduced enzyme activity by 32%. All extraction series featured the increasing activity with increasing sonication time and a plateau (data not shown).

Since enzyme activity in the non-sonicated extract is zero we conclude that the measured enzyme activity results from the effect of ultrasound upon the activated sludge flock, and not from enzymes that are possibly present in the water phase, prior to ultrasonication. We suspect that the enzyme was extracted from the extracellular polymeric matrix of the sludge flock, since mild ultrasonic conditions were applied and no cell-disruption was expected. The measured enzyme activity is thus representative for extracellular enzymes rater than intracellular enzymes. However, this conclusion would need confirmation by microscopic analysis of the non-sonicated and the sonicated sludge sample.

An increase of enzyme activity with sonication time was also observed for the fridge stored samples, but enzyme activity was significantly reduced for the fridge stored samples: Storage in the fridge during 1 and 2 d reduced enzyme activity by 30% and 87% respectively. It is therefore important to measure enzyme activity on fresh extracts, if representative results need to be obtained.

### Background Information

The occurrence of micropollutants in the aquatic environment and their fate and removal during wastewater treatment has been excellently reviewed [5]. Since micropollutants as pharmaceuticals and personal care products (PPCP) have become more and more a concern for the environment and receiving water bodies we investigate the relation between enzyme activity and micropollutant degradation in activated sludge processes. In order to do so we extracted enzymes from activated sludge samples using mild conditions in an ultrasonic bath (power determined calorimetrically) and no additives. The extraction process was thus purely ultrasonic. We reported the effect of sonication time upon enzyme activity in the enzyme extracts. It is important to establish suitable extraction conditions, i.e. ultrasonic power and sonication time, if meaningful and reproducible results are to be obtained. Storage of the enzyme extracts in the fridge and/or the freezer as well as activated sludge aeration to endogenous state prior to enzyme extraction is not recommended, since enzyme activity is significantly reduced.

## Conclusions

• Enzymes can be extracted from activated sludge using pure and mild ultrasound.
• The enzyme activity of the enzyme extracts increases with increasing sonication time.
• Storage of the enzyme extracts in the fridge drastically reduces enzyme activity.
• Aeration of activated sludge to endogenous state reduces enzymatic activity.
• Suitable extraction times need to be determined for ultrasonic enzyme extraction from activated sludge.

## References

[1] Guanghui Y.U., Pinjing H.E., Liming S.H.A.O., Yishu Z.H.U. (2009). Enzyme extraction by ultrasound from sludge flocs. J. Environ. Sci..

[2] Yu Guang-Hui, He Pin-Jing, Shao Li-Ming, Lee Duu-Jong (2007). Enzyme activities in activated sludge flocs. Appl. Microbiol. Biotechnol..

[3] Nabarlatz Debora, Vondrysova Jana, Jenicek Pavel, Stüber Frank, Font Josep, Fortuny Agustí, Fabregat Azael (2010). Christophe Bengoa Hydrolytic enzymes in activated sludge: extraction of protease and lipase by stirring and ultrasonication. Ultrason. Sonochem..

[4] Obst U. (2012). Enzymatische Tests für die Wasseranalytik München.

[5] Luo Yunlong, Guoa Wenshan, Ngo Huu Hao, Nghiemb Long Duc, Hai Faisal Ibney, Zhang Jian, Liang Shuang, Wang Xiaochang C. (2014). A review on the occurrence of micropollutants in the aquatic environment and their fate and removal during wastewater treatment. Sci. Total Environ..

